# Targeting Insulin-Like Growth Factor Binding Protein-3 Signaling in Triple-Negative Breast Cancer

**DOI:** 10.1155/2015/638526

**Published:** 2015-06-28

**Authors:** Kamila A. Marzec, Robert C. Baxter, Janet L. Martin

**Affiliations:** Hormones and Cancer Division, Kolling Institute of Medical Research, Royal North Shore Hospital, University of Sydney, St Leonards, NSW 2065, Australia

## Abstract

Insulin-like growth factor binding protein-3 (IGFBP-3) is a key regulatory molecule of the IGF axis and can function in a tissue-specific way as both a tumor suppressor and promoter. Triple-negative breast cancer (TNBC) has high tumor expression of IGFBP-3 associated with markers of poor prognosis and, although accounting for 15–20% of all breast cancers, is responsible for disproportionate rates of morbidity and mortality. Because they lack estrogen and progesterone receptors and overexpression of HER2, TNBC are resistant to treatments that target these molecules, making the development of new therapies an important goal. In addition to frequent high expression of IGFBP-3, these tumors also express EGFR highly, but targeting EGFR signaling alone in TNBC has been of little success. Identification of a functional growth-stimulatory interaction between EGFR and IGFBP-3 signaling prompted investigation into cotargeting these pathways as a novel therapy for TNBC. This involves inhibition of both EGFR kinase activity and a mediator of IGFBP-3's stimulatory bioactivity, sphingosine kinase-1 (SphK1), and has shown promise in a preclinical setting. Functional interaction between EGFR and IGFBP-3 may also promote chemoresistance in TNBC, and delineating the mechanisms involved may identify additional targets for development of therapies in cancers that express both IGFBP-3 and EGFR.

## 1. An Introduction to the Insulin-Like Growth Factor Binding Proteins in Cancer

The insulin-like growth factor (IGF) system is fundamental to normal growth and development, and by virtue of their potent proliferative and antiapoptotic effects, the polypeptide hormones IGF-I and IGF-II have also been shown to play an important role in tumorigenesis. The IGF binding proteins (IGFBPs) are key regulatory molecules of the IGF system, and aberrations in their expression or function have been associated with a range of cancers [1]. The IGFBP family comprises six IGFBPs (IGFBP-1 to IGFBP-6) that bind the IGFs with high affinity. They vary in length, ranging from 216 amino acids to 289 amino acids [2], and each consists of three regions: the highly conserved N-terminal and C-terminal domains which contain binding sites for the IGFs and the variable central or linker domain, which is also the region most commonly subject to posttranslational modification such as glycosylation, phosphorylation, and limited proteolysis and probably contributes to differences in IGFBP function [2].

The IGFBPs were first identified for their function as serum proteins that bind IGF-I and IGF-II, thereby extending the circulating half-life of IGFs from minutes to hours, regulating the hypoglycaemic potential of IGF-I and IGF-II, and controlling extravasation of the growth factors to target tissues [1]. IGFBPs also act in the pericellular environment to regulate IGF/IGF-receptor interaction, because the affinity of the IGFs for IGFBPs is similar to that for the IGF and insulin receptors [3]. This is important in the context of cancer, because IGF activation of these receptors elicits mitogenic and survival signals that promote tumor growth.

IGFBP-3 is responsible for carrying the vast majority of IGFs in the blood and is the most abundant circulating IGFBP. In this environment its role is clear: together with the acid-labile subunit (ALS), it stabilizes IGF-I and IGF-II in ternary complexes that have very slow dissociation rates and therefore long circulating half-lives [4, 5]. Release of bioavailable IGFs from these complexes is generally thought to result from limited proteolytic cleavage of IGFBP-3, which reduces its binding affinity for IGFs [6–8].

IGFBP-3 is expressed by most tissues of the body and as an antagonist of IGF binding to the signal-transducing type 1 IGF receptor (IGF1R), it blocks the proliferative and cell-survival effects elicited by its activation [3]. Consistent with this, loss of IGFBP-3 expression and consequent derepression of IGF1R signaling have been suggested to account for acquired resistance to the EGFR tyrosine kinase inhibitor gefitinib [9].* In vitro* studies in breast cancer cells have also indicated that the efficacy of some anticancer agents, including retinoic acid, antiestrogens, and tumor necrosis factor-alpha (TNF*α*), is in part mediated by IGFBP-3 [10–13]. Not all of these effects depend solely on inhibition of IGF action, and “IGF-independent” growth inhibitory or apoptotic effects of IGFBP-3 have been attributed to a variety of mechanisms, including its interaction with nuclear hormone receptors such as retinoid X receptor [14, 15] and the vitamin D receptor [16], TGF*β*/SMAD signaling pathways [17, 18], and upregulation of apoptotic effectors [19].

By contrast with these inhibitory and apoptotic effects of IGFBP-3, however, it appears that in some tissues IGFBP-3 functions as a tumor promoter as it is associated with poor patient outcomes. Overexpression of IGFBP-3 has been shown in renal clear cell carcinoma [20] and head and neck squamous cancers [21], and expression is higher in primary tumor than adjacent normal tissue or benign disease tissue in pancreatic ductal adenocarcinoma [22] and oesophageal cancer [23]. There is also evidence of IGFBP-3 being associated with metastatic disease, with IGFBP-3 expression elevated in metastatic tissue compared with primary tumor in melanoma [24], and higher in metastatic than nonmetastatic tumors in pancreatic endocrine neoplasms [25]. However, perhaps the earliest and best-documented association of IGFBP-3 with poor patient outcome is in breast cancer.

## 2. IGFBP-3 Is Highly Expressed in Aggressive Breast Cancer

Early studies investigating the expression of IGFBP-3 in breast cancer cell lines revealed a negative correlation between expression of estrogen receptor (ER) and IGFBP-3 [26, 27]. This is also seen in breast tumor tissue where many, though not all, studies have shown that expression of IGFBP-3 mRNA and protein is higher in ER-negative tumors compared to ER-positive tumors [28–31]. The clinical significance of these findings was underscored by two independent groups showing that high expression of IGFBP-3 in breast tumor tissue is associated with markers of aggressiveness and poor prognosis, including ER- and progesterone receptor- (PR-) negativity, high S-phase fraction, and aneuploidy [32, 33]. Other studies showed that while there was no significant association of high tissue IGFBP-3 protein levels with breast cancer recurrence, long-term survival was reduced [34, 35].

Both cell culture and xenograft tumor models have shown that overexpression of IGFBP-3 can indeed result in enhanced growth of breast cancer cells. T47D, an ER-positive breast cancer cell line that normally expresses low IGFBP-3, was initially growth-inhibited* in vitro* when IGFBP-3 was expressed ectopically but eventually developed resistance to its inhibitory effects and grew faster than vector-transfected cells [36]. This was reiterated* in vivo* using IGFBP-3-expressing T47D cells to establish tumors in nude mice [37], where it was found that IGFBP-3-expressing T47D tumors grew faster and larger compared to those that did not express the protein.

## 3. Mechanisms Underlying IGFBP-3's Growth-Stimulatory Actions

It seems counterintuitive that IGFBP-3, a protein that has been shown in numerous breast cancer cell studies to be growth inhibitory and proapoptotic, is associated with aggressive forms of breast cancer. However, growth-stimulatory effects of IGFBP-3 have been documented in many cell types and contexts, and a variety of mechanisms, both IGF-dependent and -independent, have been described as underlying this bioactivity. Studies in fibroblasts and mammary epithelial cells indicated that IGFBP-3 can potentiate the actions of IGFs [38–40], possibly through modulation of IGF1R activation and AKT signaling pathways [39, 40]. A “switch” in the actions of IGFBP-3 from apoptotic to antiapoptotic was shown to be dependent on the presence of matrix components, with exogenous IGFBP-3 promoting breast cancer cell survival in the presence of fibronectin, but accentuating apoptosis triggered by ceramide in the absence of fibronectin [41]. Similarly, dual effects of IGFBP-3 as both pro- and antiapoptotic molecule have been reported in human umbilical vein endothelial cells (HUVEC), with IGFBP-3 potentiating apoptosis in the presence of doxorubicin, but promoting cell survival in its absence [42]. This was shown to correlate with IGFBP-3 differentially regulating proapoptotic ceramide production by the HUVECs, with IGFBP-3 stimulating ceramide production in the presence of doxorubicin, but reducing it in the absence of doxorubicin [42].

Investigation into possible mechanisms underlying the development of resistance to IGFBP-3's growth inhibitory effects in breast cells and pathways involved in its tumorigenic bioactivity revealed that phenotypically normal MCF-10A breast epithelial cells are growth-inhibited by exogenous IGFBP-3 but become refractory to its inhibitory effects when cells express HRas, an oncogenic form of the Ras protein which is constitutively active [43]. Hs578T breast cancer cells, which also express HRas, were similarly resistant to the inhibitory effects of IGFBP-3 but were resensitized to it in the presence of PD98059, an inhibitor of p44/42MAPK [43].

Although activating Ras mutations occur relatively rarely (<5%) in breast cancer, aberrant activation of Ras signaling pathways downstream of growth factor receptors is considered an important driver of the tumorigenic process. As noted above, IGFBP-3 can enhance the growth-promoting effects of IGFs, and this has also been shown for other growth factors that activate receptors upstream of Ras, such as EGF and TGF*β*  [38, 44–47]. In MCF-10A cells, preincubation with exogenous IGFBP-3 enhanced EGF-stimulated cell proliferation, and this was associated with increased EGFR and p44/42 MAPK activation [47]. As in HRas-expressing cells, inhibition of p44/42 MAPK restored IGFBP-3's inhibitory activity [47]. Similarly, in the IGFBP-3-transfected T47D cell model described above, the acquisition of growth-stimulation associated with IGFBP-3 expression was accompanied by enhanced EGFR signaling, and pharmacological blockade of EGFR tyrosine kinase activity resensitized cells to inhibition by IGFBP-3 [37].

## 4. Potentiation of EGFR Signaling by IGFBP-3 Is Mediated by Sphingosine Kinase Signaling

Potentiation of EGF signaling by estrogen in breast cancer cells has been shown to involve transactivation of EGFR by receptors for sphingosine 1-phosphate (S1P), a bioactive phospholipid generated by the phosphorylation of sphingosine by sphingosine kinases 1 and 2 (SphK1 and SphK2) [48]. Sukocheva and coworkers showed in MCF-7 breast cancer cells that estradiol increased SphK1 activity and the formation of S1P, with subsequent binding of S1P to one of its receptors, S1P_3_, which in turn transactivated EGFR [49, 50]. IGFBP-3 had been shown to stimulate SphK1 expression and activity in HUVECs [42], raising the possibility that IGFBP-3 might enhance EGFR phosphorylation and signaling in breast cells* via* upregulation of SphK and S1P, and transactivation of EGFR. Investigation of this in MCF-10A cells showed that SphK1 expression and activity were increased by IGFBP-3 and that silencing of SphK1 expression blocked IGFBP-3's enhancement of ligand-stimulated EGFR phosphorylation [51]. The underlying mechanism involved transactivation of EGFR by S1P_1_ or S1P_3_ because pharmacological inhibition or siRNA-mediated silencing of either S1P receptor prevented the effects of IGFBP-3. IGF1R was similarly subject to transactivation by S1P receptors in cells preincubated with IGFBP-3 and stimulated with IGF-I [51]. Collectively, these data suggested that, in cells that express both IGFBP-3 and EGFR (or IGF1R), IGFBP-3 may promote growth* via *its potentiation of EGFR signaling secondary to increasing SphK1 activity and formation of S1P.

## 5. Triple-Negative Breast Cancer: A Clinical Challenge

The clinical significance of these findings in the context of breast cancer is that IGFBP-3 and EGFR are relatively highly expressed in some aggressive ER-negative and PR-negative tumors that lack amplification of HER2, which are now referred to as triple-negative breast cancers, or TNBC [31, 52, 53]. Because TNBC lack estrogen and progesterone receptors and HER2 is not amplified, these breast cancers are refractory to anticancer therapies that target these molecules. TNBC accounts for 15–20% of all breast cancer cases and often occurs in younger premenopausal women, including those of African ancestry, and is further characterized by high recurrence and high metastatic and mortality rates [53, 54]. Despite these tumors expressing EGFR, making them potentially susceptible to anti-EGFR therapies, studies have shown either no benefit or, in some instances, a worsening of clinical outcomes, associated with their use as single-line agents [55–57]. The default treatment for TNBC remains cytotoxic chemotherapy or radiation [53, 58] therapies that damage the cancer cell's DNA. The use of such therapies is associated with serious side effects [59], and the development of new treatments for TNBC that show increased efficacy but reduced toxicity is the subject of considerable interest among cancer biologists and oncologists.

## 6. Targeting IGFBP-3 Signaling through Sphingosine Kinase in TNBC

**Figure 1:**
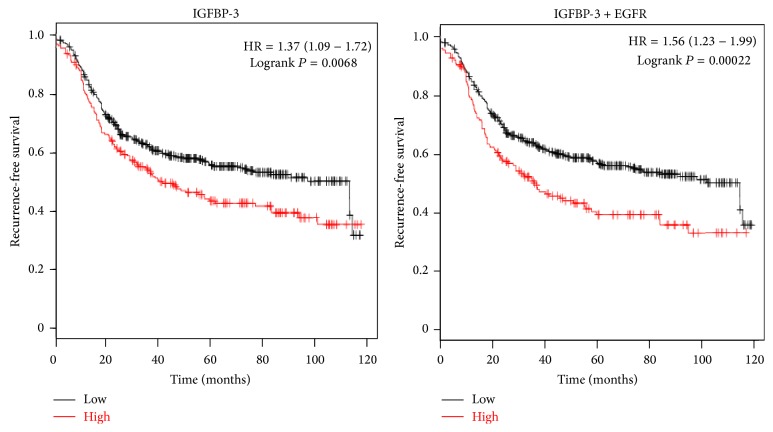
Kaplan-Meier analysis of gene expression data from 690 patients with ER-negative breast tumours shows that 10-year recurrence-free survival is significantly worse if tissue IGFBP-3 is high (red) compared to low (black), and this difference is even greater if expression of both* IGFBP3* and* EGFR* is high. HR: hazard ratio.

As alluded to above, TNBC typically express IGFBP-3 and EGFR, and Kaplan-Meier analysis of gene expression data [60] revealed that high expression of either IGFBP-3 or EGFR in ER-negative breast cancers is associated with shorter recurrence-free survival [61], and when both are highly expressed the hazard ratio is even higher (Figure 1). In light of the observed interactions between EGFR and IGFBP-3 signaling pathways, it was feasible that cotargeting these systems might be of potential benefit in the treatment of TNBC. While the most obvious way of inhibiting IGFBP-3 signaling would be to target the protein itself, its importance as an endocrine regulator of IGF metabolic activity makes such an approach unlikely to be clinically practical. An alternative approach in the setting of TNBC is to target IGFBP-3's effector pathway, the SphK system, in conjunction with EGFR inhibition.

An oncogenic role for SphK1 was first proposed more than a decade ago [62] and was supported by subsequent work showing the protective effect of SphK1 knockout against the development of tumors in a range of animal models [63–65]. Interest in SphK1 as a therapeutic target in various cancers has grown enormously [66], and a number of inhibitors of SphK have been developed, some of which have reached clinical evaluation (as reviewed in [48]). The availability of such inhibitors made it feasible that if successful, a combinatorial approach of targeting this axis with EGFR could be rapidly implemented.

As in normal breast epithelial cells, endogenous and exogenous IGFBP-3 enhanced EGF-stimulated EGFR phosphorylation in four TNBC cell lines, and this was dependent on the expression and activity of SphK1 [31]. Under conditions where inhibition of EGFR or SphK1 alone had little effect on the growth of these cell lines* in vitro*, the combination of gefitinib and SKi-II (2-(*p*-hydroxyanilino)-4-(*p*-chlorophenyl)thiazole), an inhibitor of SphK1, essentially abolished cell proliferation [31]. Importantly, when the combination was tested* in vivo* using TNBC cells grown as xenograft tumors in nude mice, gefitinib in combination with SKi-II significantly attenuated tumor growth when used at concentrations that had no significant effect as single agents [31]. These promising proof-of-principle studies indicate that the approach of cotargeting EGFR and SphK1 has potential for the treatment of TNBC. In view of the known heterogeneity of TNBC, ongoing studies are now focussing on whether all subtypes of TNBC respond similarly to this combination treatment.

## 7. IGFBP-3 and the DNA Damage Response: Can IGFBP-3 Alter Responsiveness to Chemo- and Radiotherapy?

Activation of the tumor suppressor p53 in response to chemotherapy or radiotherapy plays an important part in the cytotoxic effects of these therapies [67]. Since the discovery of* IGFBP3* as a p53-inducible gene in 1995 [68], studies in many cancer cell lines have demonstrated an increase in the expression of IGFBP-3 in response to treatment by chemotherapeutic drugs [69–71]. Because exogenous IGFBP-3 has proapoptotic activity in many cell types, including breast cancer cells, either when used alone [72] or in combination with other apoptotic agents such as C_2_ ceramide [73], chemotherapy drugs [70], or radiation [19], the induction of IGFBP-3 in response to DNA-damaging therapies has been assumed to contribute to the cytotoxicity of these treatments. Consistent with this idea, IGFBP-3 is more highly expressed in certain chemo- or radiosensitive cancer cell lines than in matched resistant cell lines, as shown in cervical carcinoma and ovarian and lung cancer cells [74–76]. Similarly, when examined in patient NSCLC tumors, loss of IGFBP-3, mediated by promoter hypermethylation, has been found to be associated with decreased chemosensitivity [77].

However, the association of high IGFBP-3 expression with poor outcome in aggressive breast cancer [32, 35, 78] may, in addition to reflecting enhanced tumor growth as described above, reflect altered responsiveness to anticancer therapies. Thus in some breast cancers high IGFBP-3 expression might be associated with treatment resistance rather than sensitivity. Skog et al. [79] speculated that the relatively high expression of IGFBP-3 in ER-negative compared to ER-positive breast cancer cells and tumor tissue [26, 29] might contribute to enhanced DNA damage repair, which might in turn lead to relative resistance to DNA-damaging therapies. Although based on a very limited study, this speculation has turned out to have some experimental support in ER-negative breast cancer cell lines.

The authors have recently reported that IGFBP-3 has an integral role in the non-homologous end-joining (NHEJ) repair response to DNA double strand breaks (DSB) caused by the topoisomerase II poisons, etoposide, and doxorubicin [80]. Of the two major mechanisms of DSB repair (homologous recombination and NHEJ), NHEJ is relatively error-prone but can occur at any stage of the cell cycle, while homologous recombination is more faithful but is generally restricted to S and G2 phase [81, 82]. NHEJ has been shown to involve EGFR which, in response to DNA-damaging agents, forms a nuclear complex with the catalytic subunit of one of the key kinases involved in NHEJ, DNA-dependent protein kinase (DNA-PKcs) [83]. In TNBC cell lines that have high IGFBP-3 expression, siRNA-mediated downregulation of IGFBP-3 was shown to inhibit the formation of this EGFR-DNA-PKcs complex [80]. Further, IGFBP-3 itself formed DNA damage-dependent complexes with both EGFR and DNA-PKcs, suggesting the possibility of a nuclear ternary complex in the DSB repair process. IGFBP-3 downregulation also directly inhibited DNA DSB repair as measured in an* in vitro* NHEJ assay [80]. This suggests a previously unrecognized involvement of IGFBP-3 in chemotherapy-induced DNA DSB repair, which may in some circumstances lead to cancer cell recovery rather than apoptosis in response to cytotoxic drugs. IGFBP-3-dependent breast cancer cell chemoresistance, while contrary to studies in some other cancers showing IGFBP-3-dependent chemosensitivity, might in part explain the association between high IGFBP-3 (and EGFR) expression and poor patient outcomes in women with ER-negative breast cancer.

## 8. Concluding Remarks

It is clear that IGFBP-3 functions as a tumor promoter in some cancers, including TNBC. In TNBC it does so, at least in part, by potentiating growth-stimulatory signaling through the EGFR, and this requires SphK1. Understanding the mechanism involved in growth-stimulatory signaling by IGFBP-3 has led to the design of a combination treatment that cotargets both pathways involved and may therefore have improved efficacy not only in TNBC but in other cancers in which EGFR is highly expressed and IGFBP-3 functions as a tumor promoter. Further dissection of the molecular interactions and pathways by which IGFBP-3 may confer chemoresistance in TNBC also has the potential to identify other targets for the development of novel therapies to treat this aggressive disease.
